# Diabetes Treatment and Mental Illness: A Call for an Integrated Health Care System in Underserved Semi-Rural Malaysia

**DOI:** 10.3390/ijerph191610015

**Published:** 2022-08-14

**Authors:** Govindamal Thangiah, Hamimatunnisa Johar, Roshidi Ismail, Ulrich Reininghaus, Till Bärnighausen, Sivakumar Thurairajasingam, Daniel Reidpath, Tin Tin Su

**Affiliations:** 1 South East Asia Community Observatory (SEACO) & Global Public Health, Jeffrey Cheah School of Medicine and Health Sciences, Monash University Malaysia, Subang Jaya 47500, Malaysia; 2 Department of Psychosomatic Medicine and Psychotherapy, University of Giessen and Marburg, 35392 Giessen, Germany; 3 Institute of Epidemiology, Helmholtz Zentrum München, 85764 Munich, Germany; 4 Department of Public Mental Health, Central Institute of Mental Health, Medical Faculty Mannheim, University of Heidelberg, 68159 Mannheim, Germany; 5 ESRC Centre for Society and Mental Health, King’s College London, London WC2R 2LS, UK; 6 Centre for Epidemiology and Public Health, Health Service and Population Research Department, Institute of Psychiatry, Psychology and Neuroscience, King’s College London, London WC2R 2LS, UK; 7 Heidelberg Institute of Global Health, Faculty of Medicine, University of Heidelberg, 69117 Heidelberg, Germany; 8 Clinical School Johor Bahru, Jeffrey Cheah School of Medicine and Health Sciences, Monash University Malaysia, Johor Bahru 80100, Malaysia; 9 Institute for Global Health and Development, Queen Margaret University, Edinburgh EH21 6UU, UK

**Keywords:** mental health, diabetes mellitus, diabetes control, blood glucose depression, anxiety, stress, glucose control

## Abstract

Diabetes mellitus (DM) management imposes a tremendous psychological burden on patients. The study investigates the association between DM treatment with blood glucose (BG) control and common mental health conditions. A cross-sectional study was conducted among 1821 individuals with DM in a community-based survey conducted in 2013. Information on respondents’ sociodemographic, mental health, DM treatment, and BG levels was collected. Multinomial logistic regression was employed to examine the association of diabetes treatment with controlled BG levels (<11.1 mmol/L) (42.5%, n = 774) or uncontrolled BG levels (34.3%, n = 625) compared with those not undergoing treatment (23.2%, n = 422) on depression anxiety, and stress. Having DM treatment and controlled BG was associated with high depressive symptoms (Relative Risk Ratio, RRR: 2.42; 95% CI 1.33–4.41) and high anxiety symptoms (1.66; 1.08–2.56) but not with perceived stress. However, treated DM with uncontrolled BG was associated with anxiety (high: 1.64; 1.05–2.56; low: 2.59; 1.10–6.09) but not depression or perceived stress. Our results suggest that being treated for DM, regardless of glucose control status, was associated with anxiety symptoms, whereas being treated with controlled BG was associated with high depressive symptoms. This situation highlights the need for integrative, multidisciplinary care for DM patients with mental health comorbidities.

## 1. Background

Diabetes mellitus (DM), especially type 2 diabetes (T2D), is a disconcerting major public health issue. Globally, 425 million people are affected by diabetes, with 80% of them located in low- and middle- income countries (LMICs) [1]. In Malaysia, the prevalence is increasing at an alarming rate; from 13.4% in 2015 to 18.3% in 2019 [2]. The prevalence was recorded among the highest in Asia Pacific [3] and is projected to continually increase the nation’s medical and societal burden [4]. Individuals with diabetes require comprehensive management that occurs both within and outside the health care system to prevent or delay complications, such as kidney failure, cardiovascular disease, amputation, or stroke [5,6]. Data from the Malaysian Diabetes Registry revealed up to 69.3% prevalence of poor glycaemic control among DM patients in primary care [7], which could be attributed to low treatment adherence and unhealthy lifestyles that further contribute to uncontrolled diabetes [8]. Furthermore, metabolic dysregulations in DM patients impose a tremendous physical and psychological burden [9]. Given the importance of health behaviours in achieving optimal glucose control in DM patients, improving the mental health state in DM patients provides an opportunity for treatment enhancements, improving their health status and lowering their risk of serious complications.

Although often neglected, mental illness, mostly characterized by depression, anxiety, and perceived stress, has been long associated with DM, especially among diagnosed patients, since the late 17th century [10]. Data from general populations and clinical settings have demonstrated that depression and anxiety are more common among diabetes patients, with prevalence surging by two-fold in DM patients compared to the general population [11,12]. Furthermore, longitudinal epidemiological studies provide strong aetiological evidence that both depression and anxiety independently increase the risk of diabetes onset [13,14]. The prognosis for comorbid depression and diabetes is worse than when each illness occurs separately [15]. The co-occurrence of mental health disorders and diabetes are also associated with adverse diabetes outcomes [16], functional disability [17], increased health care costs [18], and increased mortality [19]. Moreover, psychological distress and comorbid mental health conditions are shown to influence DM control [20,21], although this association has not been demonstrated in all studies [22,23]. Exposure to psychological or emotional stress is associated with a number of pathophysiological mechanisms that make an association between stress and diabetes risk theoretically plausible. For example, psychosocial stress can stimulate the hypothalamus–pituitary–adrenal axis, the sympathetic nervous system and inflammatory pathways, which are known to affect glucose metabolism [24,25]. 

The primary treatment goal for individuals with DM is an optimal glucose control; however, it is still unclear whether DM treatment status may differentially affect the mental health conditions of patients. Random blood glucose is routinely measured in clinical practice and included as part of the clinical practice screening guidelines in Malaysia [26], as also recommended by International Diabetes Federation (IDF) [27]. Previous large cohort studies demonstrated that random blood glucose at baseline predicted incident diabetes within five years, as well as the use of random blood glucose levels provides good discrimination for follow-up diabetes diagnosis [28]. Furthermore, random glucose elevations are more strongly associated with undiagnosed diabetes than traditional diabetes risk factors [29] and may provide an early warning sign of glycaemic dysregulation [29]. To date, no previous studies have investigated how treatment and achieving optimal blood glucose (BG) concentration in individuals with DM may affect mental health conditions in a real-world LMICs setting where the integration of mental health treatment in routine primary care, particularly in diabetes management, is still lacking. Therefore, this study attempts to examine the cross-sectional associations of DM treatment status and BG control with mental health conditions among DM patients in a multi-ethnic semi-rural adult population. Based on meta-analytic findings on the significant association between mental health impairments and diabetes treatment non-adherence [21,30] and hyperglycaemia [20], it is hypothesised that treated patients with uncontrolled diabetes are associated with mental health impairments compared to those without treatment.

## 2. Methods

### 2.1. Study Settings

The South East Asia Community Observatory (SEACO), a health and demographic surveillance system (HDSS), conducted the community-based health survey [31]. SEACO is a unique research platform that assesses the population health and well-being of semi-urban and rural population, to which most of the Malaysian population belong. It conducts annual surveys in five out of eleven sub-districts of Segamat district, Johor State, southern Malaysia. The five sub-districts were chosen based on the strong pre-existing relationship between the Jeffery Cheah School of Medicine and Health Sciences (JCSMHS) and the district as well as state health administration, which is essential to conducting this research. The demographics of Segamat comprise ethnic proportions that closely reflect the national breakdown composition of Malays (60%), Chinese (23%), and Indians (7%) [31].

### 2.2. Study Design and Participants

A cross-sectional health survey of 25,184 respondents aged 5 years and above was conducted in 2013 [31]. Of the 25,184 health survey participants, 1844 (780 males and 1064 females) participants aged 35 years and above with known diabetes mellitus were identified from the study. Only respondents who provided written consent were enrolled in this study. Using standardized health data collection tools, participants’ information on their demographic (age, gender, marital status, ethnicity), socioeconomic (income, educational level and occupation), and mental health status as well as diabetes status and treatment level were gathered. Data recorded on the tablets were then encrypted and uploaded to a secure server. The health screening and anthropometric measurements were conducted according to the World Health Organization (WHO) stepwise survey approach to the surveillance of noncommunicable diseases (STEPS) [32]. Sample enumerators were trained and briefed about the objectives of the survey. The information was recorded directly onto Android mobile devices and tablets with survey forms designed in Open Data Kit (ODK). Participants with no history of diabetes mellitus and who did not provide a random blood glucose measurement (n = 23) were excluded from the analysis. Therefore, 1821 participants with complete data on diabetes mellitus status and blood glucose levels were included in the current analysis. 

The study was approved by the Monash University Human Research Ethics Committee (MUHREC code 3837, approved on the 25 March 2013).

### 2.3. Outcome

Mental health conditions, including depression, anxiety, and stress were assessed using the validated Malay version of the Depression, Anxiety and Stress Scale (DASS-21) [33]. The questionnaire comprises of 21 items, which were divided into 3 groups each consisting of 7 items, each of these groups representing the symptoms of depression, anxiety, and stress. Participants were requested to answer questions on a 4-point Likert scale, ranging from 0 (“does not apply to me”) to 3 (“applies to me most or all the time”). Scores for each scale were summed and then classified as normal, mild, moderate, severe, and extremely severe based on the DASS manual [34]. Clinical cut-off for depression (normal 0–9; mild 10–13; moderate 14–20; severe 21–27; extremely severe ≥28), anxiety (normal 0–7; mild 8–9; moderate 10–14; severe 15–19; extremely severe ≥20), and stress (normal 0–14; mile 15–18; moderate 19–25; severe 26–33; extremely severe ≥34). These cut-off scores are derived from a set of severity ratings proposed by Lovibond and Lovibond [34]. A small proportion of 1.3%, 4.3%, and 0.4% individuals with diabetes were classified as having “severe” and “extremely severe” symptoms of depression, anxiety, and stress, respectively. In light of that, these responses were combined into the “moderate” group, which corresponded to a high level of symptoms. The final mental health variables used in this analysis consisted of three levels: “no”, “mild”, and “high” categories.

### 2.4. Main Covariates

#### 2.4.1. Assessment on Diabetes, Treatment, and BG Level

Diabetes was ascertained with a self-reported question of “Have you ever been told by a doctor or other health worker that you have raised blood sugar or diabetes?” All participants aged 35 years and above provided random blood glucose readings within the valid range of values for the glucometer. Blood glucose was estimated using a fingerprick (plasma) home monitoring glucometer (Omron model HGM-111). Due to very low prevalence of type 1 diabetes in Malaysia [7], the term “diabetes” will be used to refer to type 2 diabetes unless indicated otherwise. Participants with diabetes were then differentiated between those who were untreated, treated but with uncontrolled BG levels, and treated with controlled BG levels. Participants were classified as being treated if they answered “Yes” to either questions: (1) “Are you currently receiving insulin for your diabetes or raised blood sugar?” or (2) “Have you taken any medication in the past 2 weeks for your diabetes or raised blood sugar?” The control of diabetes was defined by random BG levels. Patients with random BG levels less than 11.1 mmol/l were defined as individuals with controlled BG levels and vice versa [26]. 

#### 2.4.2. Covariates

Covariates were chosen a priori based on their availability and previous research identifying their potential associations between diabetes treatment status and mental health conditions (anxiety, depression, and perceived stress). Previous research has shown the potential confounding role of sociodemographic and socioeconomic factors [35], as well as cardiometabolic risk factors of body mass index (BMI) [36,37] and hypertension [38,39] with mental health conditions. Sociodemographic factors included age, gender, ethnicity, marital status, and socioeconomic variables (income, educational level, occupational status). Body mass index (BMI) was calculated as weight (kg)/height2 (m^2^), which was assessed in a medical examination. Self-reported hypertension status was determined from the questions (1) “Have you ever had your blood pressure measured by a doctor or other health worker?” and (2) “Have you been told by a doctor or a health worker that you have raised blood pressure or hypertension?”.

#### 2.4.3. Statistical Analysis

Of the 25,184 health survey participants, 1821 known diabetics aged 35 and above were included in the analysis. Descriptive statistics were used to describe the characteristics of the survey respondents. Chi-square and Kruskal–Wallis tests were performed to examine whether respondents’ characteristics differed across groups. Multinomial logistic regression [40] was applied to examine the association between diabetes treatment groups with controlled and uncontrolled BG and outcome variables depression, anxiety, and stress, with the reference category of individuals who do not experience symptoms of depression, anxiety, and stress. A multicollinearity test was conducted using variation inflation factor (VIF) statistics. VIF values of less than 5.0 indicate the absence of collinearity. Descriptive and regression analyses were performed in Stata version 14.0. The significance level was set at 0.05 and 0.01.

## 3. Results

The present study includes 1821 participants with known DM (57.6% women and 42.4% men), with the majority categorised in the 50–69-years-of-age group (65.8%), of Malay ethnicity (59.5%), had primary education as the highest education level attained (55.0%), and were married (79.1%). Of the total sample (N = 1821), 23.2% (n = 422) of DM patients were not undergoing treatment and 42.5% (n = 774) of DM patients received treatment with controlled BG levels, whereas 34.3% (n = 625) were treated but had uncontrolled BG levels.

Table 1 presents the sociodemographic characteristics of the study sample by diabetes treatment categories. Statistically significant differences (*p* < 0.05) were observed between diabetes categories in age groups, ethnicity, educational level, and occupation. For example, older individuals (70 years old and above) were more likely to be undergoing treatment with controlled BG levels (22%) compared to those being treated with uncontrolled BG levels (15%) or not undergoing treatment (18%). However, middle-aged individuals (50–69 years) tend to not receive treatment or had uncontrolled diabetes. Individuals of Chinese ethnicity were more likely to be in treatment with controlled BG levels (28%) compared to those not in treatment (16%), while Malay and Indian ethnicities were less likely to be in treatment (Table 1).

As can be seen in Table 2, the proportion of participants categorized as having at least mild depression, anxiety, and stress were 13.9%, 18.2%, and 5.2%, respectively. Participants who were undergoing treatment with controlled BG levels were presented with worse mental health and cardio-metabolic profiles in comparison to other diabetes groups: they were more likely to report moderate to severe depressive and anxiety symptoms (*p* < 0.0001) and a higher prevalence of hypertension (Table 2). 

Table 3 summarizes the findings from the multinomial logistic regression analysis. In the confounders-adjusted multinomial logistic regression models calculated for each mental health condition as the outcome, being treated for diabetes with controlled BG levels was associated with having high depressive (RRR = 2.42, 95% CI = 1.33–4.41) and anxiety symptoms (1.66; 1.08–2.56). However, being treated with uncontrolled BG levels was also significantly associated with having high anxiety symptoms (1.64; 1.05–2.56). No significant association between diabetes treatment status and perceived stress was found.

## 4. Discussion

The present investigation provides evidence of an association between diabetes treatment status and the symptoms of anxiety and depression in a semi-rural community-dwelling adult population with diabetes mellitus (DM). The prevalence of treated but uncontrolled DM (34.3%) was comparable to recent data from China [41] but lower than those from Thailand [42]. Despite the notably higher prevalence of controlled DM (43.5%) than the national average (30.7%) [7], the present study also revealed that the treated with controlled diabetes status was significantly associated with high anxiety and depressive symptoms, suggesting that patients undergoing treatment, despite having optimal BG levels, are strongly linked with adverse mental health conditions.

Previous studies have consistently indicated the link between poor diabetes control and depression [20,43,44,45,46], potentially influenced by medication adherence and health-related behaviour problems [43,46]. In contrast, we found a significant association between well-controlled diabetes and depressive symptoms. Recent evidence shows that the diagnosis and treatment of diabetes most likely account for mental illness due to the awareness of having a pernicious chronic condition and its complications, including physical limitation and comorbid conditions [47]. A cross-sectional analysis of 14,328 participants in the National Health and Nutrition Examination Survey revealed that the increasing severity of depressive symptoms is associated with patients’ higher awareness of diabetes [47]. Meanwhile, the burden of dealing with a diabetes diagnosis and its complications might also lead to more severe depressive symptoms, as previously demonstrated by a population-based study among an ethnically-diverse US adult population (N = 4847) [48]. Furthermore, in a study by Bell et al., (N = 696 older adults), better foot care in DM patients was associated with elevated symptoms of depression [49], providing the link between diabetes care and mental health impairment. Of note, patients with depression were more likely to visit their primary health care physicians [50], which could explain the better diabetes control in DM patients. Interestingly, a previous study that was conducted within a Malaysian primary health clinic demonstrated that worse diabetes-related distress at baseline strongly predicted an improvement in depressive symptoms 3 years later, suggesting that depressive symptoms or more “negative feelings” at baseline could be a manifestation of initial coping behaviours in diabetes care [51]. Therefore, evidence is still inconclusive regarding the degree to which psychological distress may negatively impact glucose control in patients with type 2 diabetes, thus warranting further investigations.

Against expectations, our findings indicate that both controlled and uncontrolled diabetes treatment are associated with elevated anxiety symptoms, revealing a robust relationship between diabetes treatment and anxiety symptoms regardless of blood glucose (BG) control. To date, evidence has established the link between diabetes treatment status and depression, but less is known about anxiety symptoms. Anxiety is another common mental disorder found in patients with DM [12]. The presence of marked anxiety symptoms may lead to non-adherence to diabetes treatment [52], despite this association not being completely clear and some studies presenting contradictory results, which may be explained by the anxiety symptoms heterogeneity [20]. Several theories may explain the link between diabetes treatment status and anxiety. Individuals may often experience anxiety symptoms after being diagnosed with diabetes, suggesting the emotional impact of a diabetes diagnosis [53]. Anxiety symptoms may also lead to, or exacerbate, T2D through physiological mechanisms [54]. Furthermore, anxiety is also associated with poor metabolic control due to undesirable stress-induced lifestyle changes, which may cause individuals to lose control over their health, as well as the burden of daily diabetes management [55,56]. Moreover, a previous study highlights the bidirectionality of this relationship, demonstrating that elevated anxiety was associated with subsequent poor self-care, and poor self-care was associated with subsequent elevated anxiety after controlling for depressive symptoms as a covariate [57]. However, research has also shown that psychological factors, such as coping styles and personality, could explain the link between anxiety and diabetes control. Emotion-oriented coping strategies in people with high trait anxiety appear to benefit the long-term glycaemic control [58]. To this end, it is crucial to address anxiety and diabetes simultaneously, as both conditions may increase the patient’s risk of other potential adverse health outcomes.

Despite achieving a well-controlled BG level, the prevalence of mental health comorbidities was the highest among the controlled diabetes group. Currently, comprehensive strategies and guidelines on managing mental health issues among individuals with diabetes are still not in place in Malaysia [59], which in turn could have a bearing on diabetes treatments and blood glucose management. Therefore, it is intriguing to speculate that the lack of collaborative care might have influenced the strong link between diabetes treatment status and the mental health conditions of the present study. A multi-faceted approach to mental health management in adults with diabetes and comorbid depression is paramount [41], given findings from multiple studies showing that depression treatment improves anxiety or depressive symptomatology, although with minimal effect, if any, on diabetes self-care behaviours and glycaemic control [60,61,62]. Findings from the TEAMCare trial, which investigated the efficiency and effectiveness of the collaborative, integrated care model for diabetic and depressed patients, also showed better physical and mental health outcomes than those with usual diabetes care in primary care clinics in the US setting [63]. The implementation of a collaborative care system involves the interdisciplinary cooperation between mental and diabetes healthcare providers, routine screening, and the monitoring of health outcomes, the provision of effective treatment options, training for self-management, the supervision of care managers, and decision support for primary care physicians [64]. To date, Germany and the UK have outlined an integrated health care system that follows clinical guidelines on the treatments and medications required for diabetic patients suffering from depression, stress, and anxiety [64], the adherence to which improved collaborative care in the UK, decreased the cost of health and social care by 3.4 million in 4 years, and benefitted 11.7 million individuals due to an increase in productivity [65]. Therefore, an integrated care model in diabetes care is a promising avenue for Malaysia, given the recent acceptance of primary care clinicians for including mental health in a collaborative care effort [66] and the establishment of a diabetes register for monitoring diabetes parameters in primary care [7].

Another alternative approach to addressing mental health issues in diabetes care is by implementing social prescribing (SP) that offers alternative methods to the clinical and time-bound therapies practiced in primary care. SP is an approach that involves the referral of patients, often from a GP (General Practitioner), to a local provider of nonclinical services within the community, such as mental health facilitators, faith groups, volunteers who have experienced mental distress, or other community-based activities, such as exercise, cooking, making art, and gardening. These activities promote positive mental health and healthy lifestyles, leading to increased empowerment, self-esteem, confidence, improved mental health outcomes, cognitive functioning, and lowered feelings of social exclusion and isolation [67,68,69]. SP can occur via a link worker, who facilitates the bridge between GPs and community groups and enables greater patient access to support. Furthermore, SP enables improvements in health-related behaviours and the management of long-term conditions [70]. It has been shown that the combination of mental health and community-based exercise interventions tailored to diabetes and delivered by community mental health and exercise professionals has shown effectiveness in improving both depression and A1C values [71]. Furthermore, SP could improve the efficient use of health and social care resources by strengthening community networks and enhancing self-care [72]. Of note, health care providers may also benefit from SP by reducing the burden stemming from the lack of resources, time, and staff shortages [73]. Therefore, SP may be an effective way to address mild to moderate mental health conditions and facilitate adherence to treatment and lifestyle changes, particularly among patients with DM.

## 5. Strengths and Limitations

The strengths of this study include the assessment on the link between diabetes treatment, BG control, and mental health among semi-rural respondents, which has rarely been the focus in the literature. The study also employed a random blood glucose levels measurement for glucose monitoring, which has been shown to be a viable alternative to HbA1c measurement for community surveillance, particularly in a low-resource setting [74]. Previous meta-analysis that used the objective measures of glucose monitoring was found to show significantly stronger effects than those that relied on self-report or other methods more vulnerable to bias [21]. This study also takes into account a wide variety of socio-demographic variables, which has potential confounding effect on the association. However, this study also consists of some limitations which needs to be surmounted. Among others, this includes the lack of a longitudinal data to establish temporal priority, which will further strengthen and affirm the direction of the association between diabetes care and mental distress. Further data on diabetes management and medication adherence was also not available. The current study also used the self-reported assessment of health status (e.g., hypertension and diabetes), which has extensively been used in estimating the risks of hypertension or diabetes within populations due to convenience and cost-effectiveness. However, the accuracy of such data is a concern as self-reported hypertension assessment tends to underestimate the prevalence of hypertension among populations compared to biometric data [75,76]. Such reporting bias includes problems of recalling diagnosed and undiagnosed high blood pressure. The current study did not include the HbA1c measures to assess glucose control. It has been shown that the major setback of using HbA1c is its high cost and non-practicality [74]. While a single blood glucose measurement is inadequate for individual clinical management, it is useful for large population-based research. In the current study, we utilized a high cut-off threshold to only capture individuals with poor glucose control. Apart from that, findings from the study may not be generalizable to the Malaysian population who reside in metropolitan areas where the access to health care, including mental health services, is better than semi-rural areas. 

## 6. Conclusions

In summary, in an ethnically diverse sample of semi-rural older rural adults, depressive symptoms and anxiety symptoms are associated with diabetes treatment, particularly in individuals with optimal BG control. Thus, these findings are in line with research on psychological stress associated with diabetes management, suggesting that mental health impairments may be amplified in individuals undergoing diabetes treatment regardless of their diabetes control status. Nevertheless, the tremendous impact of untreated mental health conditions can further lead to extremely negative consequences impacting a person’s daily life. Moreover, primary care providers should develop and implement further collaborative care model strategies to address the burden of mental health impairments in individuals with diabetes. This might include routine, intensive efforts to recognize and treat mental health conditions in patients with diabetes in the primary care setting, or tailored self-management education and intervention that recognize the unique adherence barriers among adults with diabetes and depression.

## Figures and Tables

**Table 1:** Sociodemographic characteristics of study participants by diabetes status (N = 1821).

			Diabetes Categories						
	Overall		Treated But Not Controlled		Treated and Controlled		Not in Treatment		*p* *
	n	%	n	(%)	n	%			
	1821	(100)	625	(34.3)	774	(42.5)	422	(23.2%)	
Gender									
Men	772	(42.4)	259	(41.4)	327	(42.2)	186	(44.1)	0.695
Women	1049	(57.6)	366	(58.6)	447	(57.8)	236	(55.9)	
Age groups									
35 to 49	283	(15.5)	93	(14.9)	115	(14.9)	75	(17.8)	**0.015**
50 to 59	601	(33.0)	213	(34.1)	242	(31.3)	146	(34.6)	
60 to 69	598	(32.8)	226	(36.2)	248	(32.0)	124	(29.4)	
70 and above	339	(18.6)	93	(14.9)	169	(21.8)	77	(18.2)	
Ethnicity									
Malay	1083	(59.5)	362	(57.9)	463	(59.8)	258	(61.1)	<0.001
Chinese	414	(22.7)	135	(21.6)	213	(27.5)	66	(15.6)	
Indian	308	(16.9)	125	(20.0)	93	(12.0)	90	(21.3)	
Other	16	(0.9)	3	(0.5)	5	(0.6)	8	(1.9)	
Educational level									
No formal education	95	(5.3)	39	(6.3)	41	(5.3)	15	(3.6)	**0.001**
Primary	994	(55.0)	354	(57.1)	440	(57.2)	200	(47.7)	
Secondary	604	(33.4)	191	(30.8)	251	(32.6)	162	(38.7)	
Tertiary	55	(3.0)	15	(2.4)	20	(2.6)	20	(4.8)	
Other	60	(3.3)	21	(3.4)	17	(2.2)	22	(5.3)	
Marital status									
Married	1440	(79.1)	498	(79.7)	607	(78.5)	335	(79.4)	0.860
Not married	380	(20.9)	127	(20.3)	166	(21.5)	87	(20.6)	
Occupation									
Paid-employee	360	(19.8)	113	(18.1)	158	(20.4)	89	(21.2)	**0.003**
Self-employed	253	(13.9)	87	(13.9)	95	(12.3)	71	(16.9)	
Homemaker	639	(35.2)	235	(37.7)	259	(33.5)	145	(34.6)	
Not working	365	(20.1)	134	(21.5)	173	(22.4)	58	(13.8)	
Pensioners	200	(11.0)	55	(8.8)	89	(11.5)	56	(13.4)	
Income tertiles									
Lower	647	(35.5)	229	(36.6)	266	(34.4)	152	(36.0)	0.574
Middle	603	(33.1)	200	(32.0)	254	(32.8)	149	(35.3)	
Upper	571	(31.4)	196	(31.4)	254	(32.8)	121	(28.7)	

* *p*-value for differences between groups using Pearson’s χ^2^ test. Total variations due to missing data.

**Table 2:** Mental health status, BMI, and known hypertension by diabetes status (N = 1821).

			Diabetes Categories						
	Overall		Treated But Not Controlled		Treated and Controlled		Not in Treatment		*p* *
Depression	n	(%)	n	(%)	n	(%)	n	(%)	
Normal	1542	(86.0)	524	(85.8)	634	(83.2)	384	(91.6)	**0.001**
Mild	106	(5.9)	40	(6.5)	47	(6.2)	19	(4.5)	
Moderate to severe	144	(8.0)	47	(7.7)	81	(10.6)	16	(3.8)	
Anxiety									
Normal	1471	(81.6)	494	(79.9)	605	(79.1)	372	(88.8)	**0.001**
Mild	76	(4.2)	32	(5.2)	34	(4.4)	10	(2.4)	
Moderate to severe	255	(14.2)	92	(14.9)	126	(16.5)	37	(8.8)	
Stress									
Normal	1708	(94.8)	590	(95.6)	707	(92.7)	411	(97.6)	**0.003**
Mild	52	(2.9)	16	(2.6)	29	(3.8)	7	(1.7)	
Moderate to severe	41	(2.3)	11	(1.8)	27	(0.4)	3	(0.7)	
BMI, mean (SD)	28.3	(5.2)	28.7	(5.0)	28.0	(5.3)	28.4	(5.1)	**0.015 °**
Known hypertension									
Yes	1316	(72.3)	431	(69.0)	599	(77.4)	286	(67.8)	**<0.001**
No	505	(27.7)	194	(31.0)	175	(22.6)	136	(32.2)	

*p*-value for differences between groups using Pearson’s χ2 test, * for categorical variables and the Kruskal-Wallis test, ° for the continuous variable (BMI).

**Table 3:** Multinomial logistic regression model results for each DASS-21 subscale.

	Model 1: Depression ^#^				Model 2: Anxiety ^#^				Model 3: Stress ^#^			
	Mild vs. Normal		Moderate vs. Normal		Mild vs. Normal		Moderate vs. Normal		Mild vs. Normal		Moderate vs. Normal	
	RRR	(95% CI)	RRR	95% CI	RRR	95% CI	RRR	95% CI	RRR	95% CI	RRR	95% CI
Men (ref.)	-	-	-	-	-	-	-	-	-	-	-	-
Women	0.84	(0.45–1.55)	1.20	(0.72–1.98)	1.77	(0.86–3.64)	1.02	(0.67–1.54)	1.61	(0.76–3.42)	2.38	(1.01–5.65) *
Age groups												
35 to 49 (ref.)	-	-	-	-	-	-	-	-	-	-	-	-
50 to 59	1.53	(0.69–3.39)	0.87	(0.47–1.63)	0.76	(0.32–1.80)	1.39	(0.85–2.27)	1.24	(0.46–3.35)	0.53	(0.16–1.77)
60 to 69	1.91	(0.83–4.42)	1.04	(0.53–2.02)	0.92	(0.36–2.33)	1.38	(0.81–2.36)	1.44	(0.49–4.24)	0.75	(0.22–2.58)
70 and above	1.50	(0.55–4.11)	0.79	(0.35–1.80)	1.15	(0.38–3.49)	1.11	(0.57–2.16)	1.10	(0.25–4.85)	0.91	(0.22–3.68)
Ethnicity												
Malay (ref.)	-	-	-	-	-	-	-	-	-	-	-	-
Chinese	0.67	(0.38–1.18)	2.99	(1.92–4.65) ***	0.33	(0.14–0.73) **	1.30	(0.91–1.85)	5.65	(2.56–12.45) ***	2.09	(0.97–4.52)
Indian	0.49	(0.24–0.99) *	2.03	(1.19–3.45) **	0.71	(0.34–1.48)	0.76	(0.49–1.19)	3.48	(1.32–9.17) *	1.35	(0.50–3.65)
Other	3.48	(0.69–17.64)	1.79	(0.20–15.83)	8.78 × 10^−6^	(0–NA)	3.25	(0.85–12.35)	51.61	(9.87–269.94) ***	5.01 × 10^−7^	(0–NA)
Education level												
No formal education (ref.)	-	-	-	-	-	-	-	-	-	-	-	-
Primary	2.71	(0.63–11.63)	0.87	(0.40–1.91)	0.63	(0.22–1.78)	1.46	(0.69–3.08)	0.68	(0.14–3.31)	0.41	(0.14–1.19)
Secondary	1.58	(0.34–7.36)	0.40	(0.16–0.99) *	0.30	(0.09–1.03)	0.90	(0.39–2.04)	0.46	(0.08–2.51)	0.17	(0.04–0.73) *
Tertiary	0.62	(0.05–7.68)	0.37	(0.09–1.46)	0.53	(0.10–2.93)	0.38	(0.10–1.42)	0.22	(0.02–3.09)	0.20	(0.02–2.27)
Other	1.69 × 10^−6^	(0–NA)	0.40	(0.08–2.06)	0.34	(0.06–1.96)	0.43	(0.10–1.73)	5.88 × 10^−7^	(0–NA)	0.30	(0.03–2.85)
Marital status												
Married (ref.)	-	-	-	-	-	-	-	-	-	-	-	-
Not married	0.75	(0.41–1.37)	0.97	(0.58–1.61)	0.72	(0.36–1.46)	1.01	(0.68–1.52)	0.65	(0.24–1.76)	0.59	(0.24–1.43)
Occupation												
Employed (ref.)	-	-	-	-	-	-	-	-	-	-	-	-
Self-employed	0.34	(0.14–0.82) *	0.26	(0.12–0.60) **	0.64	(0.25–1.67)	0.27	(0.15–0.49) ***	0.29	(0.09–0.93) *	0.14	(0.02–1.14)
Homemaker	1.15	(0.52–2.51)	0.89	(0.46–1.70)	0.59	(0.24–1.46)	0.89	(0.53–1.51)	0.38	(0.13–1.11)	0.38	(0.12–1.22)
Not working	2.07	(1.01–4.24) *	1.26	(0.68–2.34)	1.45	(0.58–3.63)	1.29	(0.78–2.13)	0.53	(0.18–1.52)	1.01	(0.34–3.00)
Pensioners	0.07	(0.01–0.53) *	0.13	(0.04–0.45) **	0.24	(0.05–1.14)	0.07	(0.02–0.22) ***	0.11	(0.01–0.88) *	2.71 × 10^−7^	(0–NA)
Income tertiles												
Lower (ref.)	-	-	-	-	-	-	-	-	-	-		
Middle	1.05	(0.60–1.83)	0.82	(0.47–1.43)	1.11	(0.57–2.19)	0.75	(0.49–1.13)	1.80	(0.51–6.39)	0.34	(0.12–0.96) *
Upper	1.93	(1.05–3.55) *	4.02	(2.35–6.88) ***	1.84	(0.86–3.92)	2.78	(1.83–4.25) ***	10.58	(3.15–35.54) ***	1.66	(0.67–4.14)
Known hypertension												
No (ref.)	-	-	-	-	-	-	-	-	-	-	-	-
Yes	1.07	(0.64–1.78)	0.75	(0.48–1.16)	1.89	(0.93–3.83)	0.70	(0.50–0.97) *	1.53	(0.68–3.48)	1.34	(0.56–3.22)
BMI	0.99	(0.95–1.04)	0.96	(0.92–1.00)	1.02	(0.97–1.07)	0.96	(0.93–0.99) *	1.01	(0.94–1.08)	0.95	(0.88–1.02)
Diabetes group												
Not in treatment (ref.)	-	-	-	-	-	-	-	-	-	-	-	-
Treated but not controlled	1.41	(0.75–2.62)	1.72	(0.92–3.21)	2.59	(1.10–6.09) *	1.64	(1.05–2.56) *	1.74	(0.61–4.99)	1.93	(0.52–7.13)
Treated and controlled	1.21	(0.66–2.25)	2.42	(1.33–4.41) **	2.29	(0.98–5.35)	1.66	(1.08–2.56) *	1.96	(0.71–5.43)	3.34	(0.97–11.50)
n				1660				1669				1669
Likelihood ratioχ^2^				221.091 ***				214.708 ***				179.269 ***
Pseudo R^2^				0.131				0.113				0.217

* *p* < 0.05, ** *p* < 0.01, *** *p* < 0.001, ^#^ reference category of the outcome in Model 1 is no depressive symptoms, Model 2 is no anxiety, and Model 3 is no perceived stress. The regression model also revealed that, in comparison to males, females were associated with elevated stress levels. Chinese and Indian individuals, in comparison to Malay individuals, had an increased likelihood of high depressive symptoms and mild stress levels, whereas Chinese individuals had a decreased likelihood of having mild anxiety symptoms than those of Malay ethnicity. Having at least a secondary educational level significantly decreased the likelihood of high depressive symptoms and mild stress levels, whereas being in self-employment or retirement was negatively associated with perceived stress, anxiety, and depressive symptoms. Unexpectedly, higher-income was associated with adverse mental health conditions in comparison to the lower-income tertile groups, whereas unemployment (not working) was associated with mild depressive symptoms. Higher BMI and hypertension are also associated with a reduced likelihood of having high anxiety symptoms. There was no significant evidence for associations between age, marital status, and mental health impairments. NA (Not Available) was referring to data unavailability or lack of observation in the subgroup.

## Data Availability

Data are available from the SEACO study center upon reasonable request.
